# New Rat Model of Advanced NASH Mimicking Pathophysiological Features and Transcriptomic Signature of The Human Disease

**DOI:** 10.3390/cells8091062

**Published:** 2019-09-10

**Authors:** Raquel Maeso-Díaz, Zoe Boyer-Diaz, Juan José Lozano, Martí Ortega-Ribera, Carmen Peralta, Jaime Bosch, Jordi Gracia-Sancho

**Affiliations:** 1 Liver Vascular Biology Research Group, Barcelona Hepatic Hemodynamic Laboratory, IDIBAPS Biomedical Research Institute, CIBEREHD, 08036 Barcelona, Spain; 2 Barcelona Liver Bioservices, 08036 Barcelona, Spain; 3 Bioinformatics Platform, CIBEREHD, 08036 Barcelona, Spain; 4 Protective Strategies Against Hepatic Ischemia Reperfusion Injury Research Group, IDIBAPS & CIBEREHD, 08036 Barcelona, Spain; 5 Hepatology, Department of Biomedical Research, University of Bern, 3012 Bern, Switzerland

**Keywords:** steatosis, cirrhosis, hepatic fibrosis, portal hypertension, steatohepatitis

## Abstract

Non-alcoholic steatohepatitis (NASH) is a major cause of chronic liver disease. However, most available animal models fail to reflect the whole spectrum of the disease. Liver fibrosis and portal hypertension are the strongest prognostic markers in advanced NASH. We herein aimed at developing a new model of NASH in male rats, obtained using a multi-hit protocol that combines the administration of a high fat and high-cholesterol diet with CCl_4_ and phenobarbital. Following this protocol, rats showed the full characteristics of advanced human NASH after 10 weeks and NASH with cirrhosis by 24 weeks. Specifically, our NASH rats exhibited: steatosis and metabolic syndrome, lipotoxicity, hepatocellular ballooning necrosis, inflammation and importantly, marked hepatic fibrosis and significant portal hypertension. Furthermore, a whole transcriptomic analysis of liver tissue from our rat model using next generation sequencing was compared with human NASH and illustrated the similarity of this pre-clinical model with the human disease. Pathway enrichment analysis showed that NASH animals shared a relevant number of central pathways involved in NASH pathophysiology, such as those related with cell death, as well as inflammatory or matrix remodeling. The present study defines a pre-clinical model of moderate and advanced NASH that mimics the human disease, including pathophysiologic characteristics and transcriptomic signature.

## 1. Introduction

Non-alcoholic fatty liver disease (NAFLD) is considered a new epidemy in chronic liver diseases. In fact, its prevalence has been estimated to be as high as 25% in the general population and even higher (>70%) in patients with other metabolic risk factors like obesity and diabetes [1,2]. Additionally, it is well known than up to 30% of the NAFLD population will further progress and develop non-alcoholic steatohepatitis (NASH), the more severe form of fatty liver disease. NASH can progress to the end-stages of chronic liver disease, including cirrhosis and hepatocellular carcinoma (HCC) [3].

In view of the increasing incidence of NAFLD worldwide, a large number of translational laboratories are focused on understanding new aspects of NASH pathophysiology, on discovering specific and non-invasive diagnostic biomarkers, and developing effective pharmacologic treatments [4]. Nevertheless, the lack of reliable NASH animal models remains a limitation for studying the disease and for developing new therapeutic strategies [5]. Currently available animal models include genetic (Ob/Ob and Db/Db), dietary (high-fat diets [6] and methionine choline-deficient diet [7]) or a combination of both [8]. The major disadvantage of most of these models is that they fail to reflect advanced human disease. In this regard, it is important to denote that liver fibrosis represents the strongest predictor for NAFLD-specific mortality, [9] and portal hypertension is present in 25% of patients at the time of diagnosis of NASH [10]. Therefore, it is necessary to consider these pathological features to properly study NASH. Surprisingly, and despite its current relevance worldwide, no pre-clinical model of NASH with significant fibrosis and portal hypertension, together with the other relevant characteristics of the disease, has been well characterized so far [11].

Carbon tetrachloride (CCl_4_) has been widely used for decades to induce liver injury and fibrosis in murine models. Previous reports suggested that the use of CCl_4_, in combination with a high-fat diet, induced liver injury and some features of NASH at short-term [12], and HCC development at long term exposure [13]. However, these studies were developed in mice, which represents a limitation for proper hepatic hemodynamic evaluation and liver cells’ isolation. Indeed, a rat model would be preferable, because the size of the animal facilitates monitoring physiological parameters and the purification of high yields of parenchymal and non-parenchymal liver cells, and moreover, because rat models have been postulated to better correspond with human diseases [14].

Considering this background, we herein aimed at developing a new model of NASH in rats obtained using a multi-hit protocol that combines the administration of a high-fat and high-cholesterol diet with CCl_4_ + phenobarbital. Following this protocol, rats reflect the full features of advanced human NASH after 10 weeks of administration and NASH with cirrhosis by 24 weeks. We further demonstrate that this new model shares relevant pathophysiological pathways with human NASH using next generation sequencing and pathway enrichment analysis.

## 2. Materials and Methods

### 2.1. Animal Model

Male Wistar rats underwent a multi-hit liver injury protocol (termed Barcelona NASH [BarNa] model) that combines a high-fat (46.1%) and high-cholesterol (197 ppm) diet (HFCD) (Testdiet, St. Louis, MO, United States) with CCl_4_ inhalation (Sigma, Fluka, 87031) and oral phenobarbital added to the drinking water, as described previously [15]. We designed a protocol to achieve NASH after 10 weeks of intervention (NASH group) and a protocol to obtain NASH with established cirrhosis (NASH-CH group) after 24 weeks. In the NASH model, rats were exposed to 6 weeks of CCl_4_ inhalation and oral phenobarbital, followed by 4 weeks of HFCD. To obtain the NASH-CH model, rats were exposed to 8 weeks of CCl_4_ inhalation and oral phenobarbital, followed by 16 weeks of HFCD, CCl_4_ inhalation, and oral phenobarbital. Control rats were fed a standard chow diet (13% fat) (Envigo, Huntingdon, United Kingdom), with normal water for 12 weeks. *N* = 6 animals were included in each experimental group. Rats were housed (2 per cage) in a 12 h light–12 h dark cycle and were fed ad libitum. Animals were kept in environmentally-controlled animal facilities. All procedures were approved by the Laboratory Animal Care and Use Committee of the University of Barcelona and were conducted in accordance with the European Economic Community guidelines for the protection of animals used for experimental and other scientific purposes (EEC Directive 86/609).

### 2.2. Glucose Tolerance Test (GTT)

Rats were fasted for 5 h before the administration of a glucose bolus (2 mg/g, i.p.; Braun Medical, Rubí, Spain). Glycemia was determined at 0, 15, 30, 60, 90, and 120 min after glucose administration with the AccuCheck glucose sensor (Roche Diagnostics, Sant Cugat del Valles, Spain). GTT was performed 5 days before the hemodynamic studies [16].

### 2.3. In Vivo Hemodynamic Analysis

Rats were anesthetized with ketamine (100 mg/kg body weight, Imalgene 1000; Merial) plus midazolam (5 mg/kg body weight; Laboratorio Reig Jofre, S.A., Spain) intraperitoneally, fastened to a surgical board, and maintained at a constant temperature of 37 ± 0.5 °C.

A tracheotomy and cannulation with a PE-240 catheter (Portex, Ashford, UK) was performed in order to maintain adequate respiration during anesthesia. Indwelling catheters made of PE-50 polyethylene tubing (Portex, Ashford, UK) were placed into the femoral artery to measure mean arterial pressure (MAP; mmHg) and heart rate (HR; beats per minute), and to the ileocolic vein to measure portal pressure (PP; mmHg) [17,18]. Portal blood flow (PBF; mL/min) was measured with a non-constrictive perivascular ultrasonic transit-time flow probe (2PR, 2-mm diameter; Transonic Systems Inc., USA), placed around the portal vein just before its entrance in the liver, avoiding the measurement of portal-collateral blood flow. The flow probe and pressure transducers were connected to a Powerlab (4SP) linked to a computer using Chart v5.5.6 for Windows software (AD Instruments, Bella Vista, Australia). Hepatic vascular resistance (HVR) was calculated as PP / PBF. Hemodynamic data were collected after a 20-min stabilization period. Bile was collected during the stabilization period by cannulation of the bile duct with a PE-10 catheter (Portex, Ashford, UK) and expressed as bile volume (µL)/time of bile collection (min). At the end of the in vivo hemodynamic study, blood samples were collected to subsequently evaluate biochemical measurements [19]. Hemodynamic studies were performed as an end-point of the experiment after 10 weeks in the NASH group, 12 weeks in the control group, and 24 weeks in the NASH-CH group.

### 2.4. Biochemical Measurements

Liver transaminases, bilirubin, triglycerides, total cholesterol, low density lipoprotein-cholesterol (LDL), high density lipoprotein-cholesterol (HDL), and free fatty acids (FFA) were analyzed in plasma with standard methods at the hospital clinic’s CORE lab [20].

Hepatic lipid peroxidation was determined by measuring the formation of malondialdehyde (MDA) with the thiobarbiturate reaction [19].

Non-fasting plasma insulin was measured with the ultrasensitive rat insulin ELISA kit (90060, Crystal Chem, Zaandam, Netherlands), according to the manufacturer’s instructions.

### 2.5. Liver Histology

Liver samples were fixed in 10% formalin, embedded in paraffin, sectioned, and slides were stained with hematoxylin and eosin (H&E) for assessment of liver histology, and with Sirius Red for liver fibrosis evaluation. Fibrosis was evaluated as the collagen proportional area (percent of red-stained area out of total area of the liver section) using ImageJ software. NAFLD activity score and fibrosis stage were evaluated by an expert pathologist according to the NASH clinical research network (CRN) scoring system. The histological scoring was performed without knowing the rat group and protocol [21,22].

### 2.6. Oil Red O Staining

Liver samples were frozen in OCT, sectioned, and slides were stained with Oil red O (Sigma Aldrich, San Luis, MO, USA) for lipid analysis. Lipid droplets were evaluated as the red-stained area per total area using the ImageJ software [20].

### 2.7. Cell Death

Terminal deoxynucleotidyl transferase dUTP (2′-Deoxyuridine, 5′-Triphosphate) nick end-labeling (TUNEL) was performed in deparaffinized liver sections using an in situ cell death detection kit (Roche Diagnostics, Sant Cugat del Valles, Barcelona, Spain) according to the manufacturer’s instructions [18].

### 2.8. Immunohistochemistry

Liver samples were fixed in 10% formalin, embedded in paraffin, sectioned and processed for immunohistochemistry (IHC) or immunofluorescence (IF) as previously described [23]. For IHC, liver sections were incubated with antibodies against CD32b (sc-13271, Santa Cruz, CA, USA), von Willebrand factor (VWF) (A0082, Dako, Santa Clara, CA, USA), Desmin (M0760, Dako, Santa Clara, CA, USA), or CD163 (MCA342R, Biorad, Hercules, CA, USA). After incubation with corresponding secondary antibodies, color development was induced by incubation with a DAB ((3,3′-diaminobenzidine) kit (Dako, Santa Clara, CA, USA) and counterstained with hematoxylin. Sections were dehydrated and mounted. The specific staining was visualized and fifteen images per liver were acquired using a microscope equipped with a digital camera and the assistance of Axiovision software. The relative volume was calculated by dividing the number of points positive in sinusoidal areas by the total number of points over liver tissue (CD32b, vWF, and Desmin). Positive cells per field were quantified for CD163.

For IF, liver sections were incubated with antibodies against CD68 (MCA341R, Biorad, Hercules, CA, USA), myeloperoxidase (MPO) (ab9535, Abcam, Cambridge, UK), and histone H2B (ab52484, Abcam, Cambridge, UK) incubated with secondary antibodies Alexa Fluor 488 or 555 (1:400, Life technologies) and 4′,6-diamino-2-fenilindol (DAPI) (1:3000, Sigma-Aldrich, San Luis, MO, USA) and mounted in fluoromount G medium. Ten images per sample were obtained with a fluorescence microscope and positive cells per field (CD68, MPO) were quantified. Neutrophils activity was characterized as presence of neutrophil extracellular traps (NETs) (structures double-positive for myeloperoxidase and histone 2B) [24].

### 2.9. Western Blotting

Liver samples were processed, and Western blot performed as described [25]. The primary antibodies used were 3-Nitrotyrosine (N5538, Sigma Aldrich, San Luis, MO, USA), cleaved caspase-3 (9661, Cell Signalling, Danvers, MA, USA), alpha smooth muscle actin (α-SMA) (A2547, Sigma Aldrich, San Luis, MO, USA), collagen 1α1 (84336, Cell Signalling, Danvers, MA, USA), cellular retinol binding protein (CRBP1) (sc-271208, Santa Cruz), phosphorylated moesin at Thr558 (sc-12895, Santa Cruz, CA, USA), total moesin (sc-13122, Santa Cruz, CA, USA), phosphorylated endothelial nitric oxide synthase (eNOS) at Ser1177 (9571, Cell Signaling, Danvers, MA, USA), and total eNOS (610297, BD Transduction Laboratories, San Jose, CA, USA), all 1:200. Blots were revealed by chemiluminescence and protein expression was determined by densitometric analysis using the Image Studio Lite (LI-COR, Lincoln, USA). Blots were also assayed for α-tubulin (1:1000, Sigma-Aldrich, San Luis, MO, USA) content as standardization of sample loading.

### 2.10. Transcriptome Profiling

Global transcriptome profiling of rat liver tissue (*n* = 6 control, *n* = 6 NASH, and *n* = 6 NASH-CH) was performed by RNA-Seq. Briefly, 20 mg of liver tissue were weighted, and RNA was isolated using the miRNeasy mini kit (217004, Qiagen, Hilden, Germany) following the manufacturer’s instructions. The sequencing library was prepared using 1000 ng of total RNA by a TruSeq library prep kit (Illumina, San Diego, CA, USA) following the manufacturer’s protocol, and resulting data were pre-processed by our custom pre-processing pipeline. Briefly, after adapter trimming, raw sequencing reads were aligned against *Rattus Norvergicus* genome (Rnor_6.0) by Spliced Transcripts Alignment to a Reference (STAR) [26] 2-pass algorithm, and the quantification of genes and transcripts was done with the RSEM program [27] using ensembl release 95 [28]. We used the trimmed mean of M values (TMM) method and limma-voom transformation from rounded expected counts to normalize non-biological variability. An assessment of differential expression between groups was evaluated using moderated *t*-statistics [29]. The dataset is available at the National Center for Biotechnology Information (NCBI) Gene Expression Omnibus database, accession number GSE129525.

### 2.11. Bioinformatic Data Analysis

Human NASH liver transcriptome datasets were used from the GSE48452 database to identify the commonly dysregulated gene sets between human NASH and rat NASH models. See Supplementary Table S2 for patients’ clinical characteristic details: patients with NASH (*n* = 17), steatotic patients (*n* = 9) vs. healthy individuals (*n* = 12). Molecular pathway dysregulations in the rat and human liver tissues were determined by gene set enrichment analysis by means of pre-ranked Gene Set Enrichment Analysis (GSEA), in previously transformed human orthologs with the Mouse Genome Informatics (MGI) database, using the Molecular Signatures Database (MSigDB) database [30] defined as false discovery rate (FDR) <0.1.

### 2.12. Statistical Analysis

Statistical analysis was performed with the Prism 7 for Windows (GraphPad Software, San Diego, California, USA). All results are expressed as mean ± standard error of the mean (SEM). Results were compared by one-way ANOVA with Tukey post hoc test. Differences were considered significant at a *p* values of <0.05.

## 3. Results

### 3.1. BarNa Rats Develop Obesity, Liver Disfunction, and Insulin Resistance

**Figure 1:**
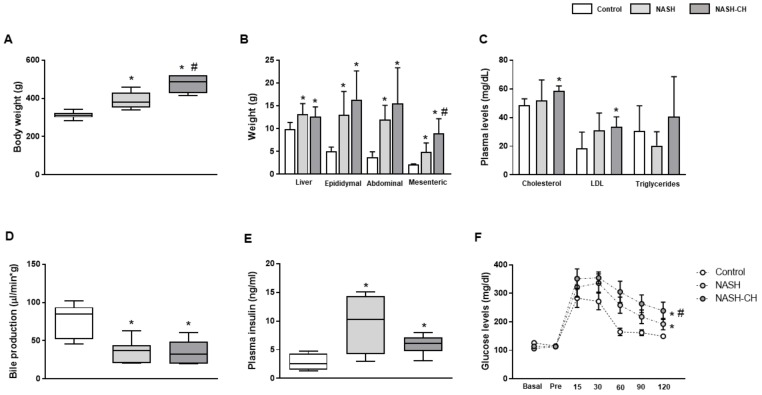
Metabolic profile of control and NASH rats. (**A**) Body weight, (**B**) liver weight, epididymal, abdominal, and mesenteric white adipose tissue weights, (**C**) plasma cholesterol, LDL, and triglycerides, (**D**) bile production, (**E**) plasma insulin levels, and (**F**) glucose tolerance tests were measured in control, NASH, and NASH-CH rats. Results represent mean ± SEM, * *p* < 0.05 vs. control group, # *p* < 0.05 vs. NASH group (*n* = 6 rats per group).

In comparison to control animals, NASH (10 weeks) and NASH-CH rats (24 weeks) showed significant body weight gain (Figure 1A), as well as an increase in liver and epididymal, abdominal, and mesenteric white adipose tissue weights (Figure 1B). Total cholesterol and LDL-cholesterol were significantly elevated in plasma from NASH-CH animals (Figure 1C), while no statistical differences were observed in the NASH group. Additionally, both models of NASH exhibited a reduction in bile production, suggesting a deterioration in hepatic functionality in comparison to control rats (Figure 1D). Plasma levels of triglycerides, HDL, FFA, and bilirubin were not altered in both groups of NASH rats (Figure 1 and Figure S1A–C). Finally, both groups of NASH animals had fasting hyperinsulinemia accompanied by an abnormal glucose tolerance test response (Figure 1E,F), features associated with insulin resistance.

### 3.2. BarNa Rats Present Liver Histology Features Distinctive of Human NASH

**Figure 2:**
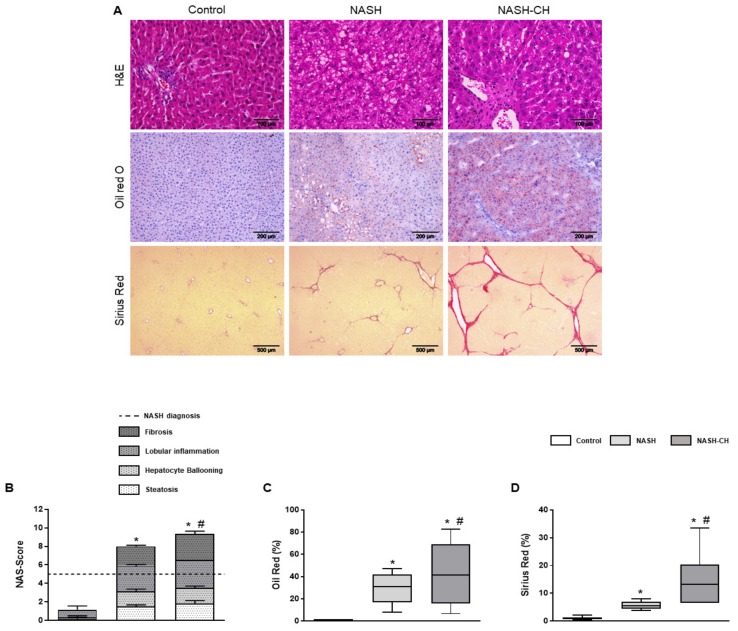
Liver histology of control and NASH rats. (**A**) Histopathological images from control, NASH, and NASH-CH livers. Hematoxylin and eosin (H&E) staining (top row; scale bar = 100 μm), oil red O staining (second row; scale bar = 200 μm) and Sirius red staining (bottom row; scale bar = 500 μm). (**B**) NAS-Score quantification. (**C**) Oil red O quantification. (**D**) Sirius red quantification. Results represent mean ± SEM, * *p* < 0.05 vs. control group, # *p* < 0.05 vs. NASH group (*n* = 6 rats per group).

All rats were ranked for each of the four histological features encompassed in human NASH (steatosis, hepatocyte ballooning, lobular inflammation, and liver fibrosis), according to the CRN Score System criteria. When compared to the control group, the NASH and NASH-CH groups exhibited a clear major scoring in all categories resulting in a final score superior to the threshold for NASH diagnosis (scores of 8 and 9, respectively) (Figure 2A,B).

Oil red evaluation revealed a significant increase in hepatic lipid deposition in NASH (30 ± 4%) and NASH-CH (43 ± 11%) rats in comparison to control animals (<1%) (Figure 2C). Furthermore, hepatic fibrosis was elevated in both NASH rats (6 ± 0.5%) and NASH-CH (15 ± 4%) compared to control rats (<1%) (Figure 2D).

### 3.3. BarNa Rats Show Elevated Oxidative Stress Levels, Cell Death, and Inflammation

**Figure 3:**
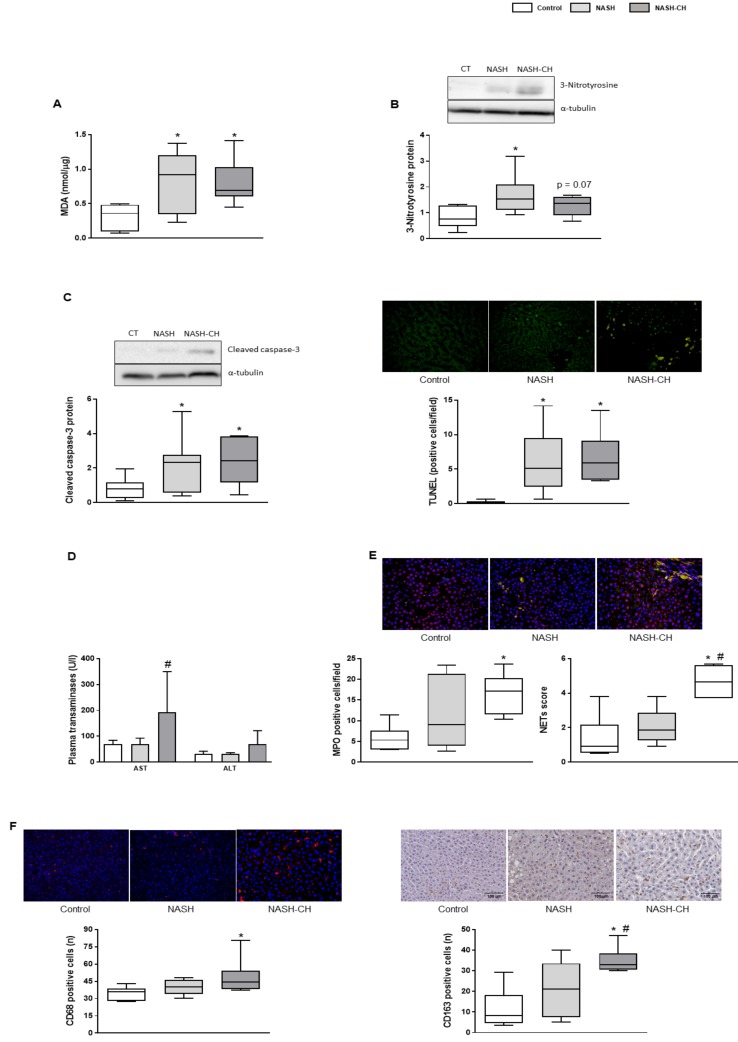
Oxidative stress, cell death, and inflammatory markers in control and NASH animals. (**A**) MDA levels in liver tissue. (**B**) Representative western blot of 3-nitrotyrosine proteins normalized to α-tubulin and quantification. (**C**) *Left*, representative western blot of cleaved caspase-3 protein normalized to α-tubulin and quantification. *Right*, representative images of TUNEL staining in liver tissue and quantification. (**D**) Plasma transaminases (AST and ALT). (**E**) Representative images of neutrophil immunofluorescence in liver tissue (MPO in green, histone 2B in red, nuclei in blue). *Left,* neutrophil infiltration measured as MPO positive cells. *Right,* analysis of hepatic neutrophil extracellular traps (NETs) determined as co-localization of MPO and histone 2B. (**F**) *Left*, representative images of CD68 immunofluorescence in liver tissue and quantification. *Right*, representative images of CD163 immunohistochemistry in liver tissue and its quantification. Results represent mean ± SEM, * *p* < 0.05 vs. control group, # *p* < 0.05 vs. NASH group (*n* = 6 rats per group). All images scale bar = 100 μm.

Rats from both BarNa models showed a significant increase in hepatic MDA levels, suggesting oxidative stress secondary to lipid peroxidation (Figure 3A). Accordingly, hepatic nitrotyrosinated proteins, marker of peroxynitrite anion formation, were elevated in NASH and NASH-CH rats compared to the control group, (Figure 3B), altogether supporting elevated oxidative stress levels in the BarNa models.

Hepatic cell death evaluation revealed significant increases in cleaved caspase-3 protein expression and TUNEL quantification in both groups of BarNa rats (Figure 3C), accompanied with elevated plasma transaminases in the NASH-CH group (Figure 3D).

NASH-CH rats presented an increase in the number of infiltrated neutrophils and in their activity (Figure 3E), together with more hepatic CD68 and CD163-positive macrophages in comparison to the control rats (Figure 3F). The NASH group had numerically higher levels of these inflammatory parameters, but they did not reach statistical significance.

### 3.4. BarNa Model Promotes the Activation and Proliferation of HSCs

**Figure 4:**
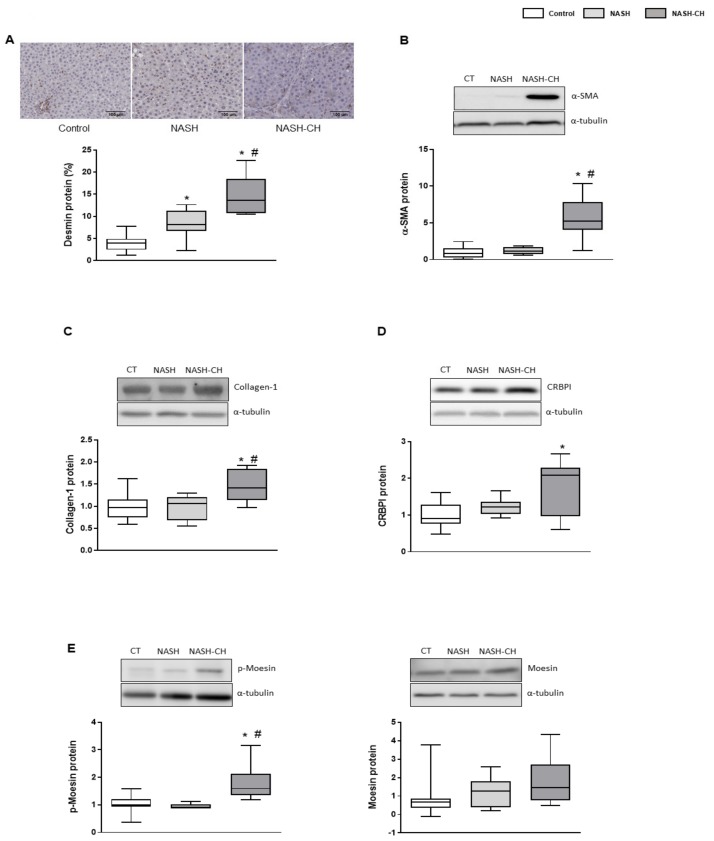
HSC phenotype in control and NASH rats. (**A**) Representative images of desmin immunohistochemistry in liver tissue and its quantification. (**B**) α-SMA protein expression in total liver tissue, normalized to α-tubulin, and corresponding quantification. (**C**) Collagen-1α1 protein expression in total liver tissue, normalized to α-tubulin. (**D**) CRBP1 protein expression in total liver tissue, normalized to α-tubulin. (**E**) p-moesin and moesin protein expression in total liver tissue, normalized to α-tubulin. Results represent mean ± SEM, * *p* < 0.05 vs. control group, # *p* < 0.05 vs. NASH group (*n* = 6 rats per group). All images scale bar = 100 μm.

BarNa rats exhibited an increase in the number of activated hepatic stellate cells (HSCs) demonstrated by the increase in desmin-positive cells and overexpression of α-SMA protein in the NASH-CH rats (Figure 4A,B). Accordingly, collagen-1α1 protein was augmented in the NASH-CH group, but not in NASH rats (Figure 4C). The expression of the chaperon CRBP1 was augmented in the NASH-CH group, suggesting a major deposition of vitamin A in these animals (Figure 4D). Additionally, HSCs from NASH-CH animals exhibited a pro-contractile phenotype as demonstrated by the increased levels of phosphorylated (active) moesin protein (Figure 4E).

### 3.5. BarNa Rats Exhibit Hepatic Endothelial Dysfunction

**Figure 5:**
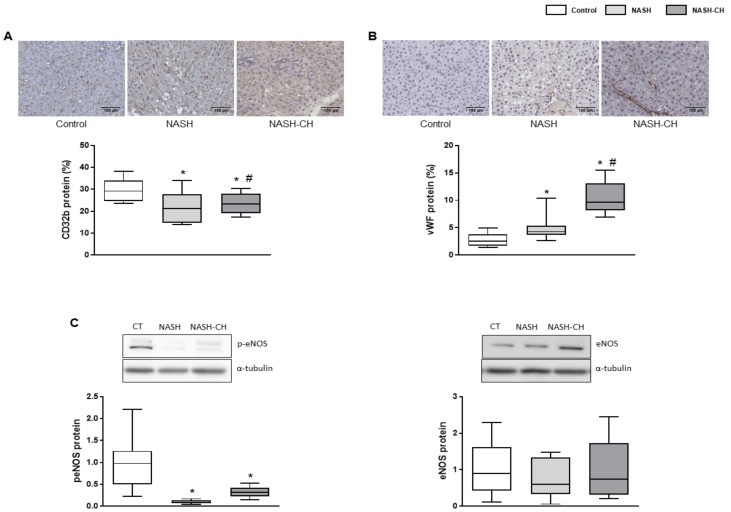
LSEC phenotype in control and NASH rats. (**A**) Representative images of CD32b immunohistochemistry in liver tissue and its quantification. (**B**) Representative images of vWF immunohistochemistry in liver tissue and its quantification. (**C**) p-eNOS and eNOS protein expression in total liver tissue, normalized to α-tubulin. Results represent mean ± SEM, * *p* < 0.05 vs. control group, # *p* < 0.05 vs. NASH group (*n* = 6 rats per group). All images scale bar = 100 μm.

Analysis of liver sinusoidal endothelial cells (LSECs) phenotype markers in NASH and NASH-CH rats suggested de-differentiation of the hepatic endothelium in comparison to the control group. NASH and NASH-CH livers exhibited reduced expression of CD32b (Figure 5A), a well-described marker of LSECs’ differentiation, as well as a remarkable increase in the sinusoidal expression of the well-established capillarization marker called the von Willebrand Factor (vWF) (Figure 5B). Moreover, LSECs from NASH and NASH-CH animals exhibited a reduction in their vasodilatory capacity as illustrated by the reduction in the active phosphorylated form of the endothelial nitric oxide synthase (eNOS) (Figure 5C).

### 3.6. BarNa Rats Show Significant Portal Hypertension

Rats from NASH and NASH-CH groups had significantly higher PP (+38% and +51%, respectively) in comparison to control animals (Table 1). The presence of portal hypertension in these animals was a consequence of a significant increase in the hepatic vascular resistance, with no evidence of hyperdynamic syndrome or presence of ascites. Systemic hemodynamic parameters showed no significant differences between groups.

### 3.7. BarNa Rats Share De-Regulations in Main Pathways Involved in Human NAFLD and NASH Pathophysiology

**Figure 6:**
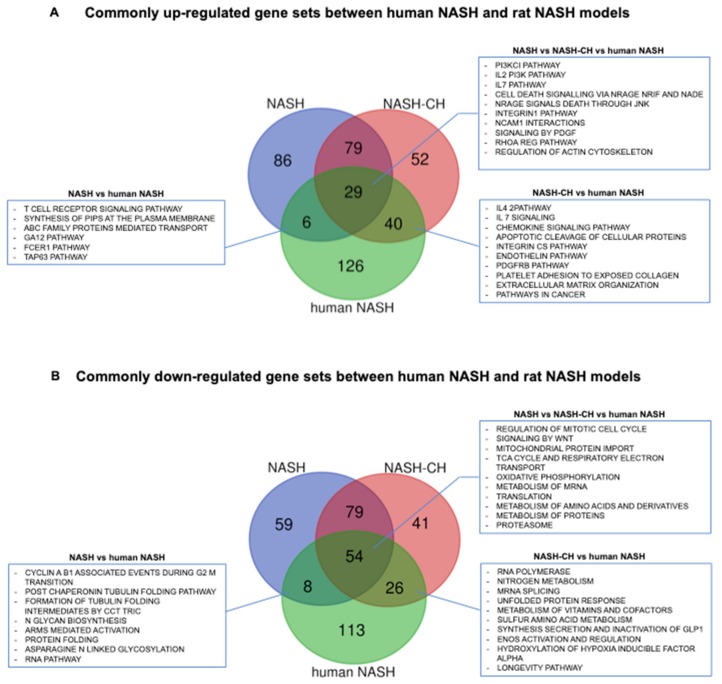
De-regulation of signaling pathways relevant for human NASH in livers from BarNa animals. (**A**) Commonly up-regulated gene sets between human NASH and BarNa rats. (**B**) Commonly down-regulated gene sets between human NASH and BarNa rats. Results represent de-regulated pathways with FDR < 0.1 in a Venn diagram. *n* = 6 animals per group; *n* = 12 control human livers, and *n* = 17 human NASH livers. Clinical characteristics of donors are described in Table S2.

Whole hepatic gene expression analysis identified 386 and 1,201 genes differentially expressed comparing NASH and NASH-CH rats with control animals at FDR <0.05 (Figure S2), respectively. Comparative transcriptomic analysis illustrated that BarNa rats share de-regulations in a significant part of pathways involved in human NAFLD and NASH pathophysiology. Pathway enrichment analysis for genes that were commonly up-regulated between NASH, NASH-CH, and human NASH showed that 29 gene sets including phosphoinositide 3-kinase (PI3K), interleukin 7 (IL7), cell death signaling, and platelet-derived growth factor (PDGF) were among the most significant dysregulated pathways (Figure 6A). 54 gene sets were commonly down-regulated between the two rat models and NASH patients and included: cell cycle regulation, metabolism of RNA, amino acids and proteins, mitochondrial regulation, and the respiratory electron cycle (Figure 6B). When comparing pathways commonly up-regulated between NASH, NASH-CH, and steatotic patients, we observed 24 gene sets significantly altered: cell death signaling pathways, coagulation, notch pathways, and basement membranes (Figure S3A). 66 gene sets were down-regulated among NASH, NASH-CH, and human steatosis including: several metabolism pathways, the cell cycle, p53 DNA damage response, and telomere maintenance (Figure S3B). Interestingly, the NASH-CH BarNa model shared more pathways with human NASH than with human steatosis, and on the contrary, the NASH BarNa model seemed to be more coincident with the dysregulated pathways in patients with steatosis.

## 4. Discussion

Nowadays, finding a reliable animal model of NASH to better understand the pathophysiology of the disease, to identify novel biomarkers and to find new targets for therapy, is still a scientific challenge. An ideal NASH animal model should mimic the full spectrum of the human disease [4]. In this regard, in the present study, we develop and characterize a new male-rat model of NASH that exhibits the major clinical characteristics of the human pathology: metabolic syndrome, hepatic steatosis, lipotoxicity, hepatic cell death, hepatic inflammation, hepatic fibrosis, and portal hypertension. Importantly, the protocol herein described is easy, affordable, and achievable in a relatively short period of time.

One important characteristic of the human disease, and absent in the most advanced NASH animal models, is the presence of obesity and metabolic syndrome [5]. Animals under the BarNa protocol presented evidence of obesity, as suggested by the body and liver weight gain and the increments in white adipose tissues. Cholesterol and LDL-cholesterol were only elevated in the most advanced model, NASH-CH, probably because these rats were fed for a longer period with the HFCD than the NASH group, which indeed agrees with the concept of Wistar rats’ resistance to developing steatohepatitis, even after long periods of time fed with a fatty diet [6]. Interestingly, BarNa rats showed insulin resistance and a moderate increase in arterial pressure, two important features associated with metabolic syndrome [31].

CCl_4_ is well-known for being a fibrogenic molecule, especially when given in combination with phenobarbital [32], which is the reason why it has been commonly used for developing animal models of liver fibrosis and cirrhosis. In our model, both groups of animals following the BarNa protocol presented significant liver fibrosis: F2–3 stage in the NASH rats and F3–4 in the NASH-CH rats. This F2–4 profile observed in our BarNa model is also present in the patients with worst prognosis [9,33], making the BarNa model attractive for studying NASH pathophysiology and anti-fibrotic therapies in advanced stages of the disease.

BarNa rats showed elevated levels of markers related with oxidative stress, cell death, and inflammation, which goes along with the natural history of NASH disease [3]. However, transaminases levels were not significantly altered in our model. Considering that AST/ALT are parameters that present high variability intra-group [13,19], we hypothesized that our sample size might not be enough to observe differences.

Activated HSCs are well known to be the cells driving fibrosis development in chronic liver disease, including NASH [34]. The NASH-CH animals present clear evidence of HSC activation and proliferation, and therefore explain the fibrotic content observed in this model. Contrarily, and although NASH animals also presented a significant increment in intrahepatic fibrosis, HSC activation markers appear to be unaltered in comparison to control animals. This discrepancy may be explained by a possible spontaneous amelioration in the HSC phenotype once CCl_4_ was discontinued and animals only received dietary intervention. Therefore, the 10-week BarNa model should be considered as a moderately advanced NASH model and could be more appropriate for studying early stages of NASH disease, while the NASH-CH BarNa model could be used for evaluating advanced fibrosis and compensated cirrhotic stages of NASH human disease.

Liver diseases, in particular those leading to fibrosis and cirrhosis, are associated with impaired vitamin A homeostasis, which may lead to vitamin A deficiency [35]. Moreover, loss of vitamin A has also been defined as a characteristic of activated HSC [36]. In NAFLD, it is not clear whether there exists a deficiency in vitamin A and/or a disturbance in its metabolism [37]. Some reports analyzing retinoid metabolism-related genes, like CRBP1, have observed an hyperdynamic state of retinoids pathway in livers from NAFLD patients [38], while others studies suggest a protective role of vitamin A in NAFLD progression [39]. Also, there is controversy regarding the contribution of stored retinoids to HSC activation [34]. In our NASH model, rats presented an inclination to higher stored levels of vitamin A. Further studies are required to fully understand the role of vitamin A in NAFLD pathophysiology.

Previous studies have demonstrated that endothelial dysfunction occurs during simple steatosis development [40] and therefore precedes the development of fibrosis and inflammation [16]. In our BarNa models, we have observed evidence of liver endothelial injury, as suggested by the expression of capillarization markers and the depletion of the vasodilatory capacity, features also recognized in the human disease [41].

Another key feature of the BarNa model, which most probably derives from the above-described pathologic events, is the presence of significant portal hypertension. This observation is relevant, since portal hypertension has been detected in almost 30% of the patients with NASH and importantly, correlates with the hepatic fibrosis stage [10,42,43]. It is well recognized that pre-clinical models of chronic liver disease do not fully mimic the broad spectrum of the human disease, and the BarNa model has limitations when compared to human NASH. Nevertheless, the presence of hepatic fibrosis and portal hypertension, together with the other important features of NASH described above, is a major strength of this pre-clinical model, since most of the currently available NASH models do not include these clinically-relevant characteristics.

In this regard, transcriptomic analysis of liver tissues using next generation sequencing reinforces the similarity of the BarNa model with the human disease. Pathway enrichment analysis illustrated that BarNa animals shared a relevant number of central pathways involved in NASH pathophysiology, including classical features of the disease (metabolism-related, inflammatory, and diabetes pathways) and characteristics of cirrhotic livers (extracellular matrix and cancer-related pathways). Although this pre-clinical model has dissimilarities at the transcriptomic level when compared to the human disease, NASH animals have more common pathways with NAFLD patients than with NASH cirrhotic patients, and on the other hand, the NASH-CH model shared more pathways with NASH patients than with NAFLD, supporting the different applicability of the two BarNa models mentioned before.

In summary, the present study defines a pre-clinical model of moderate and advanced NASH that mimics the broad spectrum of the human disease and shares a significant part of its gene signature. We herein propose the BarNa model as a reliable tool for studying the pathophysiology of NASH and for the development of new therapies.

## Figures and Tables

**Table 1:** Hemodynamic characteristics in control and NASH rats.

	Control	NASH	*p*-Value vs. Control	NASH-CH	*p*-Value vs. Control
**PP (mmHg)**	8.77 ± 0.48	12.14 ± 0.85	**<0.01**	13.26 ± 0.82	**<0.001**
**HVR (mmHg × min/mL × g)**	5.14 ± 0.79	13.14 ± 2.77	**<0.05**	12.77 ± 2.38	**<0.05**
**PBF (mL/min × g)**	1.9 ± 0.25	1.24 ± 0.25	>0.2	1.19 ± 0.22	>0.2
**MAP (mmHg)**	110→6	110→7	>0.2	112 ± 10	>0.2
**HR (BPM)**	382 ± 16	365 ± 17	>0.2	347 ± 19	>0.2

Data expressed as mean ± SEM. (*n* = 6 rats per group). PP: portal pressure; HVR: hepatic vascular resistance; PBF: portal blood flow; MAP: mean arterial pressure; HR: heart rate.

## Supplementary Materials

The following are available online at https://www.mdpi.com/2073-4409/8/9/1062/s1, Figure S1: Metabolic profile of control and NASH rats, Figure S2: Global transcriptome profiling of the BarNa liver tissue, Figure S3: De-regulation of signaling pathways relevant for human steatosis in livers from BarNa animals, Table S1: Clinical characteristics of control patients, patients with steatosis, and patients with NASH included in the gene expression and gene enrichment analysis. RNAseq data obtained from the GSE48452, Table S2: Top 10 commonly up-regulated gene sets between human NASH and BarNa rats, Table S3: Top 10 commonly down-regulated gene sets between human NASH and BarNa rats.
