# Supplementary material for: New Rat Model of Advanced NASH Mimicking Pathophysiological Features and Transcriptomic Signature of The Human Disease
Source: Cells. 2019 Sep 10;8(9):1062. doi: 10.3390/cells8091062 (PMC6770035; doi:10.3390/cells8091062)
Supplement: Supplementary file 1 [file cells-08-01062-s001.pdf]

New Rat Model of Advanced NASH Mimicking Pathophysiological Features and Transcriptomic Signature of the Human Disease

Raquel Maeso-Díaz, Zoe Boyer-Diaz, Juan José Lozano, Martí Ortega-Ribera, Carmen Peralta, Jaime Bosch and Jordi Gracia-Sancho

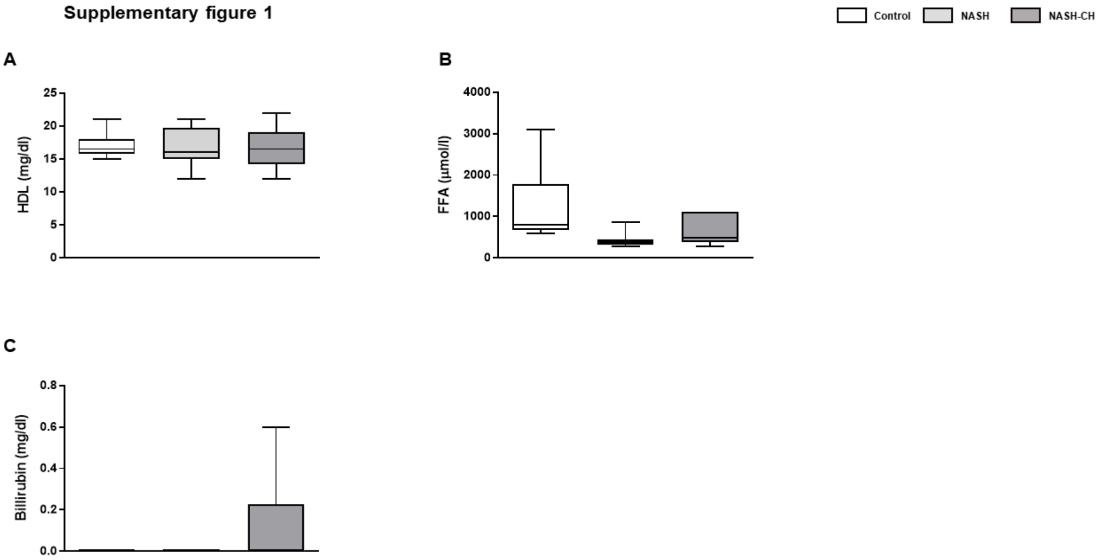

Figure 1. Metabolic profile of control and NASH rats. (**A**) HDL, (**B**) free fatty acids and (**C**) bilirubin levels were measured in plasma from control, NASH and NASH-CH rats. Results represent mean ± S.E.M. (*n*=6 rats per group).

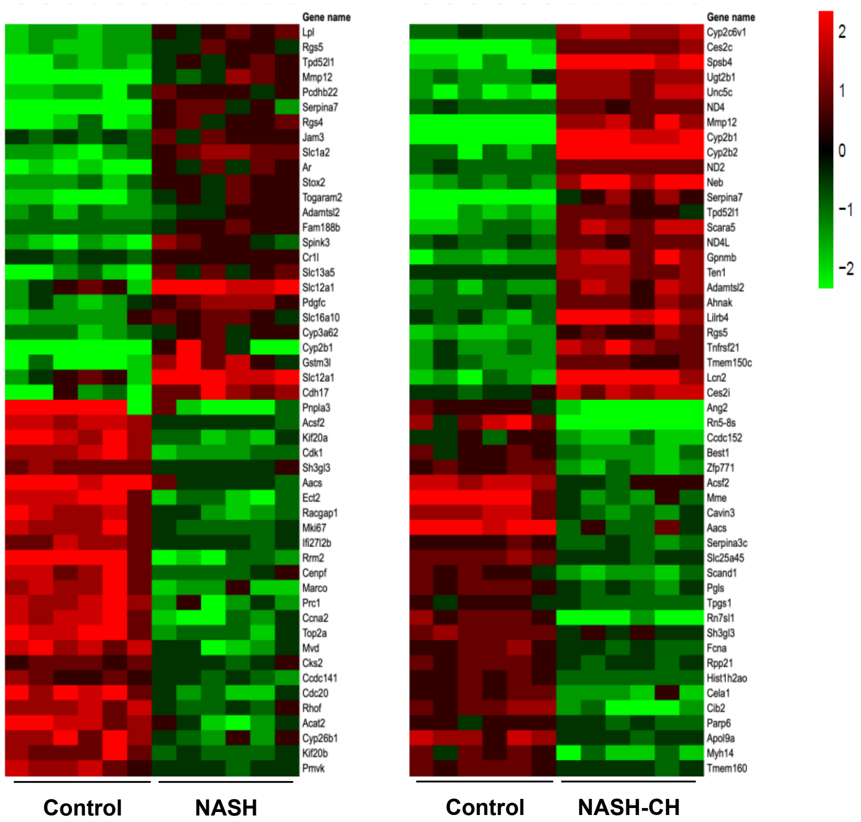

Figure 2. Global transcriptome profiling of the BarNa liver tissue. Representative image of top 50 dysregulated genes in control, NASH and NASH-CH livers. Fold enrichments (log2) are plotted in a heatmap using red colour for transcripts that are increased or using green colour for transcripts that are decreased in NASH (*left*) or NASH-CH (*right*) rat livers. FDR < 0.05, *n* = 6 per group.

Supplementary Figure 3

A Commonly up-regulated gene sets between human Steatosis and rat NASH models

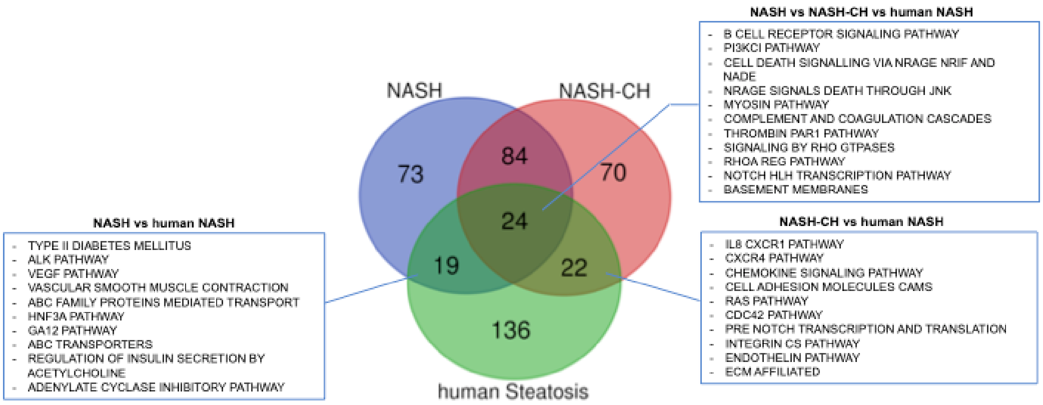

B Commonly down-regulated gene sets between human Steatosis and rat NASH models

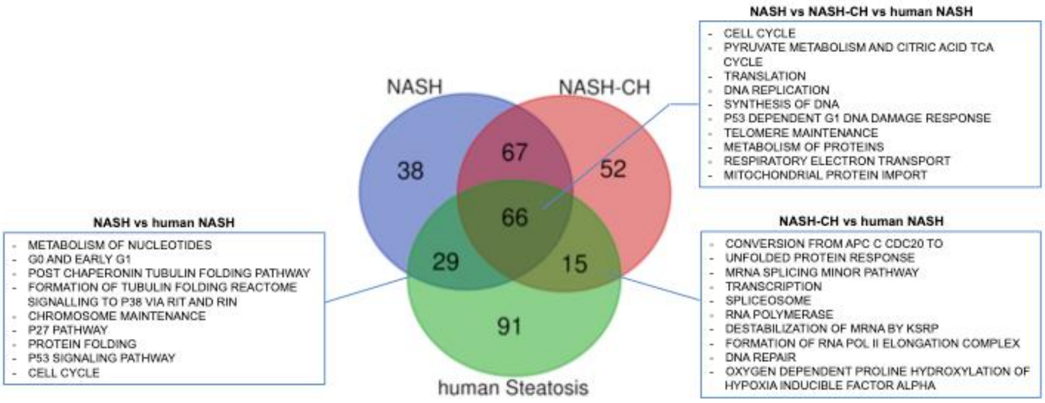

Figure 3. De-regulation of signaling pathways relevant for human steatosis in livers from BarNa animals**.** (**A**) Commonly up-regulated gene sets between human steatosis and BarNa rats. (**B**) Commonly down-regulated gene sets between human steatosis and BarNa rats. Results represent de-regulated pathways with FDR < 10% in a Venn diagram. *n* = 6 animals per group; *n* = 12 control human livers, *n* = 9 human steatotic livers. Clinical characteristics of donors are described in table S1.

Table 1. Clinical characteristics of control patients, patients with steatosis, and patients with NASH included in the gene expression and gene enrichment analysis. RNAseq data obtained from the GSE48452.

	**Controls**	**Steatosis**	**NASH**
Age	51 [44–72]	46 [37–49]	47 [40–50]
BMI	24 [21–26]	50 [47-55]	49 [44–56]
Weight (kg)	67 [58–64]	147 [121–166]	146 [133–168]
Sex (% male)	50	42	27
Diabetes (%)	11	25	20
Fat (area in %)	0 [0–1]	43 [20–70]	75 [70–85]
Inflammation (0-3)	0 [0-0]	0 [0–0]	2 [1–2]
Fibrosis (0-4)	0 [0-0]	0 [0–0]	1 [0–1]
NAS (0-8)	0 [0-0]	2 [1–3]	5 [5–6]
N	12	9	17

Table 2. Top 10 commonly up-regulated gene sets comparing human NASH and BarNa rats.

**Pathway**	**Genes**	**Database**
Signaling by PDGF	SRC, PRKCD, COL3A1, COL1A2, COL1A1, …	REACTOME
Developmental Biology	LPL, UNC5C, ANK3, PPARA, TCF4, …	REACTOME
Focal Adhesion	SRC, LAMA5, PAK1, LAMB3, TLN2, …	KEGG
Integrin1 Pathway	THBS2, ITGA9, LAMA4, LAMA5, FBN1, …	PID
Signaling By Rho GTPases	ARHGEF2, ARHGAP1, ABR, OPHN1, TAGAP, …	REACTOME
Sema4d Induced Cell Migration and Growth Cone Collapse	SEMA4D	REACTOME
Rac1 Reg Pathway	ARHGEF25, ARHGEF2, ARHGEF6, ABR, RAC1, …	PID
NCAM1 Interactions	NCAM1, COL6A1, AGRN, COL4A2	REACTOME
Semaphorin Interactions	DPYSL, PAK1, SEMA4D	REACTOME
Regulation of Actin Cytoskeleton	ITGA9, NRAS, SLC9A1, PIK3CB, SSH2, …	KEGG

PID: pathway interaction database; KEGG: Kyoto Encyclopedia of Genes and Genomes.Data expressed at FDR < 10% (n = 6 animals per group; n = 12 control human livers, n = 17 human NASH livers).

Table 3. Top 10 commonly down-regulated gene sets comparing human NASH and BarNa rats.

Pathway	Genes	Database
Citrate Cycle TCA Cycle	SDHC, SDHD, SUCLA2	KEGG
Downstream Signaling Events of B Cell Receptor BCR	PSMD12, SKP1, RPS27A, AKT1S1	REACTOME
Regulation of mRNA stability by proteins that bind Au rich elements	PSMD12, RPS271A	REACTOME
tRNA aminoacylation	TARS, FARSB, GARS	REACTOME
TCA Cycle and respiratory electron transport	SLC16A1, COX7B, COX7C, ATPSE, NDUFB1, …	REACTOME
Destabilization of mRNA by AUF1 HNRP D0	PSMD12, RPS27A	REACTOME
Nonsense mediated decay enhanced by the exon junction complex	GSPT2, RPS2, RPS15A, RBM8A, ETF1, …	REACTOME
SCDF beta TRCP mediated degradation of EMI1	PSMD12, SKP1, RPS27A, FBX05	REACTOME
Biosynthesis of the N-Glycan precursor dolichol lipid linjed to oligosaccharide LLO and transfer to a nascent protein	DPM2	REACTOME
Influenza life cycle	RPS15A, RPL31, RPL9, RPS26, RPL10, …	REACTOME

PID: pathway interaction database; KEGG: Kyoto Encyclopedia of Genes and Genomes. Data expressed at FDR < 10% (n = 6 animals per group; n = 12 control human livers, n = 17 human NASH livers).
